# Structural design dictates flavor release kinetics in potato chips: Unraveling the mechanism by integrated temporal sensory and chemical analysis

**DOI:** 10.1016/j.crfs.2026.101432

**Published:** 2026-05-02

**Authors:** Ying Wang, Jitian He, Fankui Zeng, Xianping Li, Wenjing Huang, Yuanxing Liu, Chunyan Zhang, Qiongfen Yang, Wanlin Yang

**Affiliations:** a Industrial Crops Research Institute, Yunnan Academy of Agricultural Sciences, Kunming, 650200, China; b Yunnan Technology Innovation Center of Potato(Under Preparation), Kunming, 650200, China; c Research Center for Natural Medicine and Chemical Metrology, Lanzhou Institute of Chemical Physics, Chinese Academy of Sciences, Lanzhou, China; d Yunnan Academy of Grain and Oil Sciences, Kunming, 650033, China; e Yunnan LiShi Industrial (Group) Co., Ltd, Zhaotong, 657000, China; f Shandong Hanon Scientific Instruments Co., Ltd, Dezhou, 251500, China; g Horticultural Research Institute of Yunnan Academy of Agricultural Sciences, Kunming, 650200, China

**Keywords:** Potato chips, Structural morphology, Oral processing, Retronasal aroma, GC-IMS, Flavor release kinetics

## Abstract

Although food morphology is crucial to sensory perception, its role in controlling dynamic flavor release remains inadequately explored. This study reveals that corrugated structures in potato chips function as physical micro-reservoirs, enabling a sustained-release effect for key aroma compounds. Using an integrated approach (GC-IMS, E-nose, TDS), we demonstrated that corrugated chips, with their superior mechanical strength, slowed oral breakdown, thereby delaying the T_max_ and prolonging the release of compounds such as hexanal and 2-methylbutanal. This engineered release profile perceptually translated into enhanced crispness and prolonged flavor dominance (p < 0.05), suggesting a sustained-release effect. Aldehydes and esters were pinpointed as potential key aroma active compounds by ROAV analysis. Our findings establish a clear structure–release–perception linkage, bridging physical structure to olfactory perception, and offer a mechanistic basis for optimizing sensory properties via morphological design.

## Highlights

• Chip morphology was engineered (flat, fine-, and deep-corrugated) to modulate flavor release.
• GC-IMS, E-nose & TDS revealed morphology's critical role in aroma kinetics and sensory dominance.
• Corrugated structures slowed oral breakdown, providing physical encapsulation and sustained release of key aromas.
• A non-linear relationship was found between chip hardness and the total release of key flavor compounds.
• Morphology design prolonged crispness perception and enhanced flavor persistence (TDS).

## Graphical abstract

**Figure:**
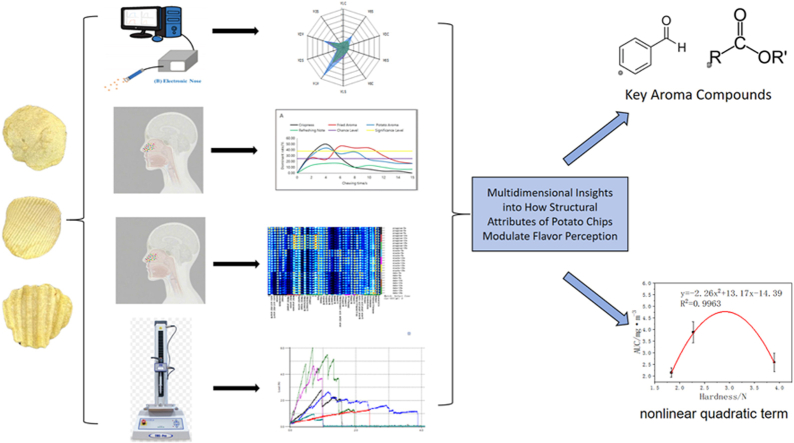

## 1 Introduction

Aroma perception during food consumption is a dynamic process, occurring through both orthonasal (from the environment) and retronasal (from the oral cavity) pathways (Pu et al., 2022). For solid foods, the structural breakdown during chewing is a critical driver of retronasal aroma, as it controls the release of volatile compounds from the food matrix. These released aroma molecules then travel through the retronasal pathway to reach the olfactory receptors, a process heavily influenced by the food's initial structure and its real-time destruction during mastication (van Eck et al., 2021).

Potato chips, as a globally popular snack, present an ideal model system where morphology dictates the efficiency of this sensory delivery. Prior work has established links between chewing effort, crispness, and overall flavor perception in chips (Luckett et al., 2016; Luckett and Seo, 2017). However, a critical gap remains in quantitatively connecting specific morphological features to the dynamic sequence of aroma release and, subsequently, to the evolving sensory experience during consumption.

This gap is primarily due to methodological limitations. Traditional analytical methods like GC-MS lack the temporal resolution to track the rapid, transient release of volatiles during chewing. Furthermore, robust predictive models remain underdeveloped for elucidating how geometric features of chips, including corrugation wavelength and amplitude, influence key oral processing mechanics such as fracture behavior and saliva integration, which in turn shape the retronasal aroma profile. The emergence of GC-IMS offers a promising solution for real-time VOCs monitoring with high time-resolution, as shown in studies on solid food oral processing (W. He et al., 2020; Wang et al., 2020). Beyond its established history in fields like security (Borsdorf and Eiceman, 2006) and medicine (Chaim et al., 2003), the application of GC-IMS has been extended to food science, where it has begun to play a pivotal role. Recent studies have successfully linked physical breakdown to aroma release in products such as rice cakes (Asimi and Min, 2025) and bread (Pu et al., 2019, 2020). Despite this progress, its application to complex, porous-brittle matrices like potato chips is not yet systematic, especially in tandem with temporal sensory methods like TDS to bridge morphology, release kinetics, and perceptual dominance.

To tackle this, we developed an integrated platform combining GC-IMS and TDS. Our goal is to clarify how chip morphology mechanistically influences flavor release. The GC-IMS component captures the real-time kinetics of VOCs release, while TDS maps the temporal competition of key sensory attributes (crispness, fried aroma, potato aroma, freshness) throughout the mastication cycle. Under strictly controlled simulated oral conditions, we compared three distinct chip morphologies: flat, fine-corrugated, and deep-corrugated. The parameters were selected to reflect typical human oral physiology: temperature was set at 37 °C to match oral temperature, salivary flow rate at 1.5 mL/min to approximate unstimulated whole saliva secretion, and chewing frequency at 1.5 Hz, which falls within the natural range (1.3-1.7 Hz) reported for crispy foods such as potato chips. We anticipate that the outcomes of this work will establish a theoretical foundation and offer practical insights for designing chip structures that enhance flavor delivery and consumer satisfaction.

## 2 Materials and methods

### 2.1 Raw materials

The potato cultivar used was 'Yunshu 304′, bred by the Industrial Crops Research Institute, Yunnan Academy of Agricultural Sciences (Kunming, China). This cultivar is characterized by its high zinc content (12.3 ± 0.8 mg/kg DW) and low reducing sugar content (0.28 ± 0.03%), making it the first dedicated, zinc-rich processing variety for potato chips in China. All tubers were harvested at 140 days after planting and stored at 10 °C and 85% relative humidity prior to processing.

### 2.2 Potato chip processing

Potato chips were manufactured under standardized industrial conditions by Yunnan Zhaotong Lishi Food Co., Ltd. (China). The manufacturing procedure was as follows: Firstly, the potato tubers were mechanically sliced into specific geometrical shapes using different types of cutting blades. A study was conducted to examine the impact of slice thickness (1.50 ± 0.05 mm, 2.48 ± 0.05 mm, 3.50 ± 0.05 mm) and surface topography (flat, fine corrugated (wavelength 3.0 ± 0.04 mm, amplitude 2.48 ± 0.01 mm), deep corrugated (wavelength 6.0 ± 0.05 mm, amplitude 4.0 ± 0.01 mm)) on flavor release dynamics. The geometrical parameters of all blades were verified using a laser profilometer (Keyence VHX-7000, Japan).

**Fig. 1:**
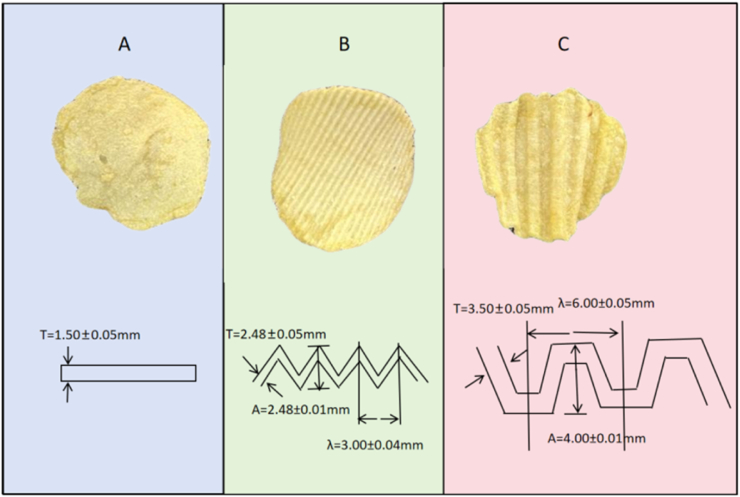
Schematic diagrams of potato chips with different slice morphologies: (A) Flat, (B) Fine corrugated, and (C) Deep corrugated. The key geometrical parameters illustrated are thickness (T, mm), and, for the corrugated slices, amplitude (A, mm) and wavelength (λ, mm), respectively.

Subsequently, the slices were fried in palm oil at 180 ± 0.5 °C for 150 s until the moisture content and fat content reached 1.8 ± 0.2% and 34.5 ± 0.7%, respectively. After frying, the chips were centrifuged for de-oiling (300 rpm, 20 s) and cooled to 25 °C at ambient temperature. A schematic diagram of the final chip morphologies is presented in Fig. 1.

### 2.3 Experimental design

This study aimed to investigate the impact of three distinct geometrical morphologies on the flavor release dynamics of potato chips. These three specific morphologies were defined as follows: (1) 1.50 mm thickness with a flat blade slice, (2) 2.48 mm thickness with a fine corrugated blade slice, and (3) 3.50 mm thickness with a deep corrugated blade slice. To minimize potential confounding effects, all other processing parameters (e.g., frying time/temperature, centrifugal force, raw material batch) were strictly maintained constant. Immediately after production, all chip samples were packaged in nitrogen-flushed aluminum-laminated pouches and stored in the dark at 20 °C. All analyses were completed within 7 days of sample preparation.

### 2.4 Sensory evaluation

TDS was employed to investigate the dominant sensory perceptions during the entire oral processing of potato chips. Ten trained panelists (5 females and 5 males, aged 20-40 years) participated in this study. All panelists were screened to have no history of dental diseases, rhinitis, salivary dysfunction, or mastication impairments, were non-smokers, and had no specific preference for potato chips. Prior to the experiment, written informed consent, detailing the research objectives, procedures, and sample information (non-toxic and harmless), was provided and signed by all participants. The study was approved by the Academic Committee of the Institute of Economic Crops, Yunnan Academy of Agricultural Sciences.

The TDS analysis comprised three stages: panel training, attribute selection, and formal evaluation. During training, panelists were familiarized with a series of reference standards for both aroma and texture until they could consistently identify each characteristic. Furthermore, following the method of Sokolowsky et al. (Sokolowsky et al., 2015), specialized training was conducted to help panelists understand the concept of "sensory dominance" — recording the attribute that most captures their attention at a specific moment, rather than its mere intensity (Watanabe et al., 2019). Panelists first individually recorded the perceived sensory descriptors when consuming the samples. Through subsequent group discussions, four TDS attributes and their definitions were finalized: Crispness (texture), Fried aroma, Potato aroma, and Freshness (aroma, characterized by a light, non-greasy sensation). All panelists were required to fast for at least 2 h before the sessions. For each test, 2.0 ± 0.1 g of potato chips was evaluated. From the moment the sample was placed in the mouth until swallowing (within 15 s), panelists continuously selected the dominant attribute from the list presented on a screen. Each panelist performed the TDS evaluation in triplicate, resulting in 30 assessments in total (10 panelists × 3 replicates). All sessions were conducted in a dedicated sensory laboratory maintained at 25 °C and 50% relative humidity. The dominance rate, defined as the proportion of time each attribute was selected as dominant over the total number of evaluations, was calculated.

Based on the dominance rates at different mastication time points, TDS curves were plotted, including the "chance level" and "significance level." The chance level, representing the probability of an attribute being selected randomly, was calculated as 1/p, where p is the number of attributes. The significance level, indicating the minimum threshold for an attribute's dominance rate to be significantly higher than others (p < 0.05), was determined according to the binomial distribution model described by Pineau et al. (2009)

### 2.5 E-nose analysis

The volatile profile of the potato chip samples was analyzed using a PEN3 electronic nose (AIRSENSE Analytics, Germany). Following the method described by Zhao et al. (Zhao et al., 2025)with modifications, 10 g of intact chips from each morphology were weighed and immediately transferred into a 100 mL glass beaker, which was then sealed with two layers of preservative film and allowed to equilibrate at room temperature (25 °C) for 20 min to allow for headspace accumulation.

Measurement was initiated by inserting the instrument's needle through the film seal into the headspace of the sample vial. The analysis was performed under the following operational parameters: a sample intake flow rate of 400 mL/min, a measurement time of 60 s per sample, and an automatic sensor flushing period of 300 s between successive analyses to prevent cross-contamination. Each sample was measured in triplicate (n = 3).

### 2.6 Collection and analysis of aroma during oral and retronasal processing

To capture the volatile organic compounds released via the oral and retronasal routes during mastication, this study employed Teflon® sampling bags to collect exhaled breath from participants, and analysis was performed using gas chromatography-ion mobility spectrometry based on the method of Pu et al. (2020) with modifications. During mastication, the panelists inhaled exclusively through the nose. At predetermined chewing time points (5, 10, and 15 s), the participants immediately exhaled the oral air into a sampling bag. This oral air sample was collected to represent the background aroma from the oral cavity (e.g., residual saliva or prior food residues) and was used as a blank control in subsequent data processing. For retronasal aroma, participants exhaled through the nose with lips tightly sealed into another bag, thereby capturing volatile compounds that migrated from the oral cavity to the nasal cavity. To prevent cross-contamination, fresh potato chip samples were used for each sampling, and participants thoroughly rinsed their mouths before each new measurement. In the subsequent data analysis, the volatile signals obtained from the chip samples were corrected by subtracting the corresponding oral background signals at each time point, ensuring that the results accurately reflected the aroma released from the potato chips themselves. Each participant placed 2.0 ± 0.1 g of potato chip sample into the mouth and chewed at a standardized frequency of 1.5 Hz guided by a metronome. At chewing times of 5 s, 10 s, and 15 s, the bolus was expectorated into a pre-weighed plastic cup. One potato chip was chewed per time point per participant, resulting in a total of nine chips per participant. Accordingly, 90 chips were prepared per chip type: 3 chewing time points × 10 participants × 3 replicates. The collected bolus was transferred into a 50 mL centrifuge tube and diluted with 45 mL of anhydrous ethanol. Gentle and continuous stirring was performed using a plastic dropper to disperse the particles until a homogeneous dispersion was achieved. After filtration, the particles were dried, spread evenly on white paper, and analyzed for particle size using Nano Measurer 1.2 software.

### 2.7 GC-IMS analysis

Volatile compounds were analyzed using a gas chromatography–ion mobility spectrometry system (FlavourSpec®; G.A.S., Dortmund, Germany) equipped with an MXT-WAX capillary column (30m × 0.53 mm i.d., 1.0 μm film thickness; Restek, Bellefonte, PA, USA). Data processing was performed using VOCal software (version 0.4.10, G.A.S.).

GC conditions:

Injection volume: 1 mL via a heated loop (80 °C).

Oven temperature: 60 °C (isothermal).

Carrier gas: high-purity nitrogen (≥99.999%).

Flow program: 2.0 mL/min (held for 2 min), increased linearly to 10.0 mL/min over 8 min, then to 100.0 mL/min over 10 min, and finally to 150.0 mL/min over another 10 min.

Total run time: 30 min.

IMS conditions:

Ionization source: tritium (^3^H, 6 keV).

Drift tube length: 53 mm; temperature: 45 °C.

Electric field strength: 500 V/cm.

Drift gas: high-purity nitrogen (≥99.999%) at a flow rate of 75 mL/min.

Detection mode: positive ion mode.

### 2.8 Determination of Relative Odor Activity Values (ROAV)

The contribution of individual volatile compounds to the overall aroma profile during oral processing was assessed by calculating the ROAV according to the method described by Zhao et al. (Y. Zhao et al., 2025). The ROAV was calculated using the following equation:

ROAV=(Ci/Ti)×100

where:

Ci is the relative concentration of the compound i obtained from GC-IMS analysis (expressed as mg/L).

Ti is the orthonasal odor threshold of compound i (mg/L in air), sourced from a book named“Odor thresholds complications of odor threshold values in air, water and other media” (Gemert, 2011).

A ROAV value greater than or equal to 1 (using 100 for the reference compound) indicates that the compound is a potential key contributor to the flavor perception during mastication.

It is important to note that this calculation relies on orthonasal odor thresholds obtained under static conditions, whereas the present study focuses on dynamic oral and retronasal release. Therefore, ROAV is employed here as a qualitative screening tool to identify candidate aroma compounds, rather than as a quantitative measure of their sensory impact during dynamic mastication.

### 2.9 Release kinetics of key aroma compounds

Key release kinetics parameters were derived from the time-series GC-IMS data collected at 5, 10, and 15 s. The parameters calculated for each significant aroma compound were:

AUC (Area Under the Curve): Representing the total amount of the compound released over the entire 15-s monitoring period. A higher AUC indicates a greater total release.

C_max_ (Maximum Concentration): The peak concentration of the compound detected during the monitoring window, reflecting its maximum release intensity.

T_max_ (Time to Maximum Concentration): The time point at which C_max_ was reached. A higher T_max_ suggests a slower release rate and a more delayed perception of that compound.

The AUC was calculated using the trapezoidal rule method. The determination of C_max_ and T_max_ was performed by identifying the maximum value in the concentration-time profile and its corresponding time point. The methodology for these calculations was adapted from established procedures in Guo et al. (郭倩倩, 2024). and van Eck et al. (van Eck et al., 2021).

### 2.10 Puncture test of potato chips

The textural properties of the potato chips were determined using a TMS-Pro Food Texture Analyzer (FTC, USA). Chips of similar size and morphology were selected for analysis. A puncture test was performed using a spherical probe with a diameter of 12.7 mm. The test parameters were set as follows: a 25 N load cell, a test speed of 30 mm/min, a puncture distance of 10 mm, and a trigger force of 0.1 N. From the resulting force-displacement curves, the key parameters of hardness (maximum force, N) and fracturability (force at the first significant break, N) were extracted.

### 2.11 Statistical analysis

Unless otherwise stated, all experiments were conducted with three independent biological replicates. Data are presented as mean ± standard deviation (SD). Statistical analysis and graphing were performed using OriginPro software (OriginLab Corp., USA). A significance level of p < 0.05 was applied to all tests.

GC-IMS Data: The relative peak volumes of volatile compounds from different chip morphologies were compared using one-way ANOVA. The kinetic parameters (AUC, Cmax, Tmax) of key aroma compounds were compared among the three morphologies using a one-way ANOVA followed by Tukey's HSD test.

TDS Sensory Data: TDS curves were constructed by calculating the dominance rate for each attribute at every second. The chance level and significance level were determined according to the method described by Pineau et al. (2009). The chance level was defined as 1/k (where k is the number of attributes, k = 4, resulting in a chance level of 0.25). The significance level (p < 0.05) for each time point was calculated using a binomial proportion test.

Texture Data: The hardness and fracturability values of the three chip morphologies were compared using a one-way ANOVA. Where significant differences were found, Tukey's HSD test was applied for pairwise comparisons.

Correlation Analysis: The relationships between textural parameters and flavor release kinetics were initially examined using Pearson's correlation coefficient. Subsequently, based on observed trends in the data suggesting a non-linear relationship, a polynomial (quadratic) regression model was employed to fit the relationship between hardness and the AUC of specific compounds. The goodness-of-fit for the regression model was evaluated using the coefficient of determination (R^2^).

## 3 Results and discussion

### 3.1 Morphology-driven regulation of aroma release and sensory perception

Initial assessment of the volatile profile of intact chips using electronic nose technology revealed that the influence of chip morphology on sensor response patterns was not significant (Table 1). To ensure the reliability of this conclusion, we further performed GC-IMS analysis on intact, uncrushed chips, and the results showed that there were no significant differences in volatile flavor compounds among the three morphologies, with all samples clustering together in the PCA plot (Fig. 3 ). This indicates that under tightly controlled processing conditions, including harvest time, frying time, and temperature, the effect of morphology on the initial aroma profile was minimized, ensuring that subsequent differences in dynamic release can be attributed to the regulation of release behavior by morphology during oral processing.

**Table 1:** Aroma signal profiles from different sensors of the E-nose.

	A	B	C
W1C	0.99 ± 0.00a	0.99 ± 0.00a	0.99 ± 0.00a
W5S	1.74 ± 0.09b	1.75 ± 0.62a	1.74 ± 0.11b
W3C	1.00 ± 0.00a	1.00 ± 0.00a	1.00 ± 0.00a
W6S	0.98 ± 0.01a	0.98 ± 0.01a	0.98 ± 0.00a
W5C	1.00 ± 0.00a	1.00 ± 0.00a	1.00 ± 0.00a
W1S	1.21 ± 0.29a	1.21 ± 0.26a	1.21 ± 0.15a
W1W	1.54 ± 0.01a	1.54 ± 0.00a	1.54 ± 0.01a
W2S	1.02 ± 0.00a	1.02 ± 0.00a	1.02 ± 0.00a
W2W	1.80 ± 0.00a	1.80 ± 0.00a	1.80 ± 0.00a
W3S	0.92 ± 0.00a	0.92 ± 0.00a	0.92 ± 0.00a

The same letter in the same row indicates no significant differences in the content of volatile compounds in intact potato chips before oral processing (P < 0.05). The sensor response values are expressed as mean ± standard deviation.W1C: Aromatic compounds, Benzene compounds; W5S: Nitrogen oxides; W3C: Ammonia, Aromatic compounds; W6S: Hydrogen; W5C: Alkanes, Aromatic compounds; W1S: Short-chain alkanes; W1W: Inorganic sulfides; W2S:Alcohols; W2W: Aromatic compounds, Organic sulfides; W3S: Long-chain alkanes.

**Fig. 2:**
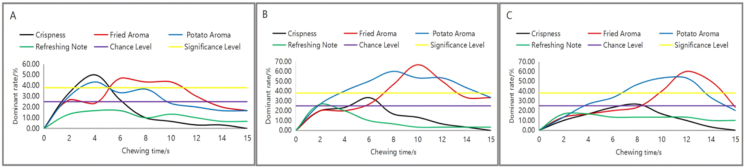
Temporal dynamics of aroma perception during the oral processing of potato chips.

**Fig. 3:**
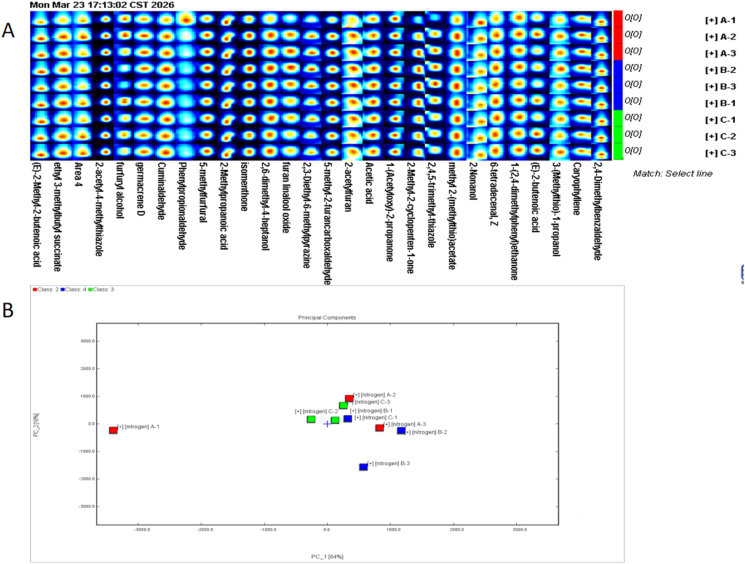
GC-IMS analysis of potato chips with different slice morphologies: (A) flavor fingerprints before oral processing; (B) principal component analysis of volatile compounds before oral processing.

This structural variation was directly translated into dynamic sensory perception. Temporal Dominance of Sensations (TDS) analysis revealed that chip morphology reconfigured the temporal narrative of perception (Fig. 2). The simple structure of flat chips produced a sequential profile: an initial dominance of ‘Crispness’ during rapid fracture, followed by a mid phase transition to ‘Fried Aroma’. In contrast, corrugated structures fostered a competitive sensory environment, where ‘Potato Aroma’ and ‘Fried Aroma’ rivaled ‘Crispness’ from the outset. Notably, the time to reach peak dominance for ‘Fried Aroma’ increased with structural complexity—from 6 s for flat chips to 10 s for fine-corrugated and 12 s for deep-corrugated chips. The sustained dominance of this attribute in deep-corrugated chips at 15 s (23.3%) compared to flat chips (16.7%) provided strong perceptual evidence of a sustained release effect, prompting a targeted investigation into the underlying release kinetics.

### 3.2 Sustained-release kinetics and key aroma contributions elucidated by GC-IMS

**Fig. 4:**
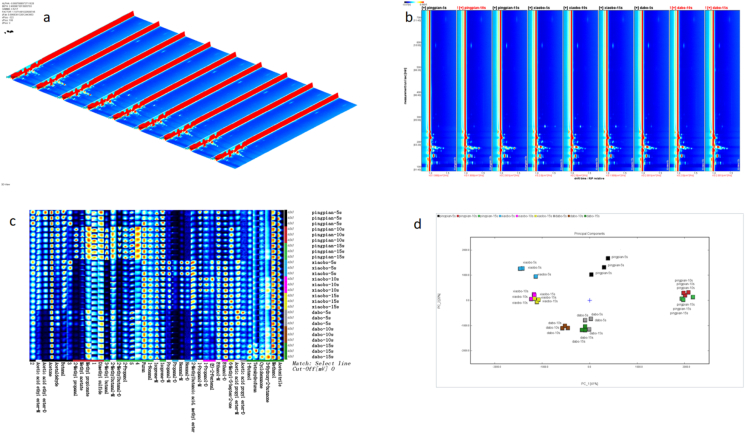
GC-IMS flavor fingerprints of potato chips with different slice morphologies during oral processing. a:Three-dimensional topographic of volatile compounds of different slice morphologies. b:Two-dimensional GC-IMS spectrum of the potato chips. c: volatile flavor compound profiles of potato chips with different slice morphologies during oral processing. Each row represents all selected signal peaks from a sample, while each column indicates the differences in signal intensity of the same compound across different samples. darker color signifies a higher compound concentration and stronger signal intensity. "-M″ and "-D″ denote monomer and dimer, respectively. Section A illustrates the variations in volatile flavor compounds of flat chips during oral processing. Section B displays the variations in volatile flavor compounds of fine rippled chips during oral processing. Section C presents the variations in volatile flavor compounds of deep rippled chips during oral processing. d: Principal component analysis of volatile compounds in potato chips with different slice morphologies during oral processing.

To decipher the chemical basis of the prolonged flavor perception, we employed GC-IMS to track the real-time release of volatile organic compounds (VOCs) during oral processing. A total of 36 VOCs were identified (Table 2, Fig. 4), and their release dynamics were profoundly governed by chip morphology. In the data analysis, the volatile signals at each time point were corrected by subtracting the corresponding oral background signals, thereby eliminating potential interference from the oral cavity itself and ensuring that the reported release profiles accurately reflected the aroma compounds originating from the potato chips.

**Table 2:** The volatile compounds during potato chips oral processing detected by GC-IMS.

N.O.	Compound	CAS	Retention index	Retention time	drift time	Odor characteristics
1	Acetone	C67641	817.5	143.851	1.11901	fresh, apple, pear
2	2-Propanol	C67630	916.2	171.875	1.2179	alcohol, spicy
3	Isoprene-M	C78795	674.7	111.384	1.06467	oil
4	Isoprene-D	C78795	689.9	114.46	1.22019	oil
5	Propanal-M	C123386	784.7	135.649	1.06467	pungent, green grassy
6	Propanal-D	C123386	786.1	135.99	1.14644	pungent, green grassy
7	Methyl acetate	C79209	802.7	140.091	1.19775	Ester, Green
8	2-Methyl propanal	C78842	794.5	138.041	1.27791	banana, melon, slightly nutty
9	Ethanol-M	C64175	934.9	178.71	1.04383	aromaticity
10	Ethanol-D	C64175	934	178.368	1.1256	aromaticity
11	Methanol	C67561	897.1	165.886	1.02909	alcohol, pungent
12	Acetic acid ethyl ester-M	C141786	893.9	164.935	1.10448	fresh, fruity, sweet, grassy
13	Acetic acid ethyl ester-D	C141786	889.7	163.713	1.3357	fresh, fruity, sweet, grassy
14	3-Methyl butanal	C590863	928.9	176.481	1.39968	chocolate, fat
15	Dimethyl sulfide	C75183	766.2	131.222	0.95113	cabbage, sulfur, gasoline
16	Acetaldehyde	C75070	748.8	127.188	1.09214	green, slight fruity
17	Furan	C110009	856.9	154.366	0.94037	special
18	2-Methylbutanal-M	C96173	921.8	173.901	1.16385	almond, cocoa, malt
19	2-Methylbutanal-D	C96173	918.3	172.627	1.39808	almond, cocoa, malt
20	Acetic acid propyl ester-M	C109604	982.8	197.47	1.16385	fruity, pear
21	Acetic acid propyl ester-D	C109604	983.8	197.895	1.47575	fruity, pear
22	1-Propanol-M	C71238	1044.1	229.957	1.11485	alcohol, pungent
23	1-Propanol-D	C71238	1043.1	229.32	1.25228	alcohol, pungent
24	Acetonitrile	C75058	1020.6	216.368	1.03717	floral
25	1-Hexanal	C66251	1095.4	262.65	1.25986	fresh, green, fat, fruity
26	1-Hexanal	C66251	1094.4	261.932	1.55682	fresh, green, fat, fruity
27	(E)-2-Pentenal	C1576870	1133.4	295.283	1.11622	potato, peas
28	1-Butanol	C71363	1149.8	310.703	1.18077	wine
29	6-Methyl-5-hepten-2-one	C110930	1349.5	586.364	1.17655	citrus, fruity, mouldy, ketone
30	3-Hydroxy-2-butanone	C513860	1294.6	489.86	1.06188	butter, cream
31	Cyclohexanone	C108941	1292.8	487.042	1.15557	strong pungent, earthy
32	1-Nonanal	C124196	1399.4	690.616	1.4688	rose, citrus, strong oily
33	Methyl propionate	C554121	925.6	175.281	1.32939	Fruit, Rum
34	2-Methylbutanoic acid, methyl ester	C868575	994	202.153	1.18212	apple
35	Butanal	C123728	879.9	160.864	1.09736	pungent, fruity, green leaf
36	Tetrahydrofuran	C109999	862.5	155.935	1.06082	ether
37	1	∗	838.8	149.451	1.15515	
38	2	∗	984.3	198.107	1.09095	
39	3	∗	1004.4	207.45	1.07063	
40	4	∗	916.5	171.987	1.25648	
41	5	∗	929.7	176.802	1.27449

The data for key aldehydes provided compelling evidence for a physical encapsulation mechanism. Hexanal, a key lipid oxidation marker (J. He et al., 2021; Zamora et al., 2007), exhibited a stable concentration profile in deep-corrugated chips throughout mastication (0.65-0.63-0.65, p > 0.05), in stark contrast to its declining trend in flat chips (Fig. 7, Table 3). Similarly, the release of (E)-2-Pentenal, characterized by a potato-like aroma (Xu et al., 2023; Zhang et al., 2025), was delayed in fine-corrugated chips, peaking at 10 s (Fig. 7, Table 3). This behavior is consistent with corrugated structures acting as physical micro-reservoirs, where the complex three-dimensional architecture retards the fracture-driven release of encapsulated volatiles (Mao et al., 2017; Yang et al., 2016; Bayarri et al., 2006; Ćorković et al., 2021).

**Table 3:** Differences in volatile flavor compound concentrations of potato chips with different slice morphologies during oral processing.

	A-5s	A-10s	A-15s	B-5s	B-10s	B-15s	C-5s	C-10s	C-15s
(E)-2-Pentenal	0.26 ± 0.05bcd	0.22 ± 0.02cd	0.16 ± 0.01d	0.39 ± 0.09 ab	0.44 ± 0.05a	0.27 ± 0.01bcd	0.32 ± 0.03abc	0.25 ± 0.10bcd	0.21 ± 0.01cd
1-Butanol	0.24 ± 0.02bc	0.20 ± 0.02bc	0.19 ± 0.04bc	0.19 ± 0.01bc	0.20 ± 0.01bc	0.22 ± 0.05bc	0.24 ± 0.01 ab	0.17 ± 0.00c	0.31 ± 0.02a
1-Nonanal	0.61 ± 0.01 ab	0.64 ± 0.04 ab	0.68 ± 0.05a	0.56 ± 0.04b	0.68 ± 0.02a	0.64 ± 0.02 ab	0.62 ± 0.04 ab	0.70 ± 0.07a	0.71 ± 0.06a
1-Propanol	1.34 ± 0.30 ab	1.12 ± 0.08bc	1.01 ± 0.07bc	1.11 ± 0.07bc	1.51 ± 0.01a	1.00 ± 0.06bc	1.24 ± 0.19abc	1.03 ± 0.03bc	0.91 ± 0.01c
2-Methyl propanal	0.11 ± 0.01d	0.41 ± 0.02a	0.37 ± 0.00b	0.07 ± 0.00e	0.06 ± 0.00e	0.06 ± 0.00e	0.14 ± 0.01c	0.07 ± 0.00e	0.06 ± 0.01e
2-Methylbutanal	1.72 ± 0.20c	3.07 ± 0.06a	3.27 ± 0.02a	1.34 ± 0.06d	1.39 ± 0.07d	1.28 ± 0.08d	2.03 ± 0.04b	1.74 ± 0.02c	1.80 ± 0.06bc
2-Methylbutanoic acid, methyl ester	0.33 ± 0.05e	0.42 ± 0.01cd	0.40 ± 0.00cde	0.79 ± 0.03a	0.69 ± 0.01b	0.75 ± 0.02 ab	0.46 ± 0.04c	0.36 ± 0.02de	0.39 ± 0.01cde
2-Propanol	1.74 ± 0.13bc	2.53 ± 0.06a	2.57 ± 0.04a	1.34 ± 0.06d	1.40 ± 0.03d	1.40 ± 0.04d	1.92 ± 0.07b	1.72 ± 0.03c	1.86 ± 0.05bc
3-Hydroxy-2-butanone	0.33 ± 0.02cd	0.38 ± 0.00bcd	0.42 ± 0.05abcd	0.32 ± 0.01d	0.48 ± 0.04 ab	0.45 ± 0.04abc	0.37 ± 0.03bcd	0.47 ± 0.04 ab	0.53 ± 0.11a
3-Methyl butanal	0.98 ± 0.19c	2.72 ± 0.11b	3.10 ± 0.05a	0.54 ± 0.04de	0.38 ± 0.02e	0.43 ± 0.02de	0.96 ± 0.17c	0.64 ± 0.04de	0.66 ± 0.02d
6-Methyl-5-hepten-2-one	0.76 ± 0.10c	1.47 ± 0.12a	1.33 ± 0.03 ab	0.95 ± 0.07bc	1.31 ± 0.07 ab	1.48 ± 0.03a	1.28 ± 0.17 ab	1.45 ± 0.27a	1.18 ± 0.16 ab
Acetaldehyde	0.41 ± 0.05a	0.35 ± 0.01 ab	0.30 ± 0.02bc	0.27 ± 0.03c	0.16 ± 0.01d	0.16 ± 0.02d	0.29 ± 0.02bc	0.15 ± 0.00d	0.19 ± 0.01d
Acetic acid ethyl ester	2.83 ± 0.41a	2.08 ± 0.05bc	1.82 ± 0.07c	2.47 ± 0.14 ab	2.14 ± 0.01bc	2.04 ± 0.04bc	1.12 ± 0.06d	0.89 ± 0.02d	1.03 ± 0.03d
Acetic acid propyl ester	0.71 ± 0.14cd	0.67 ± 0.05cd	0.60 ± 0.03d	1.06 ± 0.07 ab	0.87 ± 0.03bc	0.88 ± 0.03bc	1.24 ± 0.13a	0.82 ± 0.06c	0.77 ± 0.03cd
Acetone	27.12 ± 1.59a	22.66 ± 0.11a	15.35 ± 13.10a	23.89 ± 0.80a	21.99 ± 0.10a	22.84 ± 0.17a	22.38 ± 0.55a	23.05 ± 0.55a	22.82 ± 0.13a
Acetonitrile	19.49 ± 3.34a	20.61 ± 0.43a	14.17 ± 12.09a	17.58 ± 1.55a	21.52 ± 0.51a	22.48 ± 1.06a	21.31 ± 0.66a	24.05 ± 0.52a	23.23 ± 0.23a
Butanal	1.03 ± 0.12a	0.92 ± 0.04 ab	0.54 ± 0.46abc	0.94 ± 0.01abc	0.93 ± 0.05abc	0.89 ± 0.03 ab	0.42 ± 0.02abc	0.32 ± 0.01c	0.40 ± 0.01bc
Cyclohexanone	0.19 ± 0.03a	0.19 ± 0.03a	0.12 ± 0.10a	0.17 ± 0.00a	0.18 ± 0.01a	0.18 ± 0.03a	0.19 ± 0.03a	0.16 ± 0.01a	0.21 ± 0.04a
Dimethyl sulfide	2.67 ± 0.15a	2.79 ± 0.07a	1.84 ± 1.57a	1.93 ± 0.08a	1.92 ± 0.07a	1.92 ± 0.06a	2.60 ± 0.15a	2.51 ± 0.05a	2.54 ± 0.07a
Ethanol	10.81 ± 0.24 ab	8.81 ± 0.06 ab	5.74 ± 4.90b	14.15 ± 0.44a	15.08 ± 0.23a	13.84 ± 0.19a	15.33 ± 0.67a	15.19 ± 0.18a	14.63 ± 0.13a
Furan	2.08 ± 0.25a	2.08 ± 0.07a	1.43 ± 1.22a	2.05 ± 0.30a	1.96 ± 0.07a	1.97 ± 0.08a	2.17 ± 0.25a	2.23 ± 0.05a	2.03 ± 0.05a
Hexanal	0.68 ± 0.05 ab	0.67 ± 0.05 ab	0.37 ± 0.32b	2.55 ± 0.19a	1.71 ± 0.08a	1.75 ± 0.07 ab	0.65 ± 0.03 ab	0.63 ± 0.04 ab	0.65 ± 0.02 ab
Isoprene	5.72 ± 0.29a	5.18 ± 0.05a	3.60 ± 3.07a	5.58 ± 0.25a	4.90 ± 0.01a	4.78 ± 0.04a	4.12 ± 0.29a	4.70 ± 0.04a	4.83 ± 0.03a
Methanol	10.42 ± 0.49a	10.38 ± 0.23a	7.02 ± 5.99a	9.72 ± 0.62a	10.16 ± 0.02a	10.45 ± 0.22a	10.51 ± 0.81a	11.02 ± 0.21a	11.00 ± 0.14a
Methyl acetate	1.16 ± 0.04a	2.45 ± 0.10a	1.51 ± 1.29a	1.07 ± 0.05a	0.84 ± 0.02a	0.81 ± 0.01a	1.50 ± 0.04a	0.88 ± 0.01a	0.81 ± 0.03a
Methyl propionate	0.51 ± 0.06a	0.69 ± 0.04a	0.44 ± 0.37a	0.38 ± 0.01a	0.40 ± 0.01a	0.37 ± 0.02a	0.59 ± 0.04a	0.45 ± 0.03a	0.61 ± 0.03a
Propanal	1.83 ± 0.06 ab	2.07 ± 0.06 ab	1.27 ± 1.09b	5.09 ± 0.22a	3.70 ± 0.08a	3.75 ± 0.13 ab	2.06 ± 0.12 ab	1.74 ± 0.02 ab	1.82 ± 0.06 ab
Tetrahydrofuran	0.80 ± 0.05a	0.79 ± 0.02a	0.55 ± 0.47a	0.78 ± 0.03a	0.82 ± 0.02a	0.78 ± 0.02a	0.80 ± 0.05a	0.80 ± 0.02a	1.17 ± 0.17a
1	0.35 ± 0.02a	0.39 ± 0.02a	0.24 ± 0.20a	0.29 ± 0.01a	0.22 ± 0.01a	0.22 ± 0.02a	0.25 ± 0.02a	0.21 ± 0.01a	0.21 ± 0.01a
2	0.22 ± 0.01a	0.15 ± 0.01 ab	0.10 ± 0.08b	0.17 ± 0.02 ab	0.15 ± 0.01 ab	0.13 ± 0.01b	0.17 ± 0.02 ab	0.13 ± 0.00b	0.13 ± 0.00b
3	0.73 ± 0.12a	0.31 ± 0.02c	0.23 ± 0.19c	0.68 ± 0.16 ab	0.36 ± 0.03c	0.31 ± 0.01c	0.45 ± 0.08bc	0.31 ± 0.01c	0.32 ± 0.02c
4	0.20 ± 0.02a	0.27 ± 0.01a	0.18 ± 0.15a	0.16 ± 0.01a	0.16 ± 0.00a	0.16 ± 0.01a	0.22 ± 0.01a	0.18 ± 0.01a	0.21 ± 0.00a
5	1.61 ± 0.14a	2.31 ± 0.03a	1.56 ± 1.33a	1.42 ± 0.04a	1.28 ± 0.03a	1.33 ± 0.02a	2.05 ± 0.07a	1.71 ± 0.02a	1.77 ± 0.03a

Different letters in the same row means the significant differences of contents in potato chips caused as oral processing (P < 0.05). The contents were expressed as the average ± standard deviation.

This phenomenon was further quantified through kinetic parameters (Table 9). For methyl propionate, the time to reach maximum concentration (Tmax) showed a tendency towards delay, with the peak occurring at 15 s in deep-corrugated chips compared to 10 s in flat chips, suggesting a slower release profile. Crucially, this delayed release was achieved without a reduction in the total amount released (comparable AUC), demonstrating that morphology can strategically reprogram the timing of flavor delivery without sacrificing its overall impact.

ROAV analysis, using 2-methylbutanal as the reference (ROAV = 100), identified 13 compounds with ROAV≥1 as candidate aroma compounds (Table 4), predominantly aldehydes and esters. Given the qualitative nature of this screening tool, these compounds were further evaluated based on their dynamic release behavior. Chip morphology strategically modulated their release; for instance, the ROAV of 2-methylbutanal and 3-methylbutanal increased sharply in the later stages of chewing in flat chips, indicating a "delayed enhancement," whereas their release in fine-corrugated chips was lower and stable over time (p > 0.05). When integrated with the TDS results showing prolonged fried aroma dominance in corrugated chips, these dynamic release patterns suggest that morphological design can influence the temporal profile of key aroma compounds.

**Table 4:** The ROAVs of volatile compounds detected by GC-IMS during the oral processing of potato chips.

Compounds	Threshold mg/L	ROAV								
		A-5s	A-10s	A-15s	B-5s	B-10s	B-15s	C-5s	C-10s	C-15s
(E)-2-Pentenal	0.0005	6.01	2.82	1.99	11.63	12.75	8.51	6.33	5.72	4.78
1-Butanol	0.01	0.27	0.13	0.12	0.28	0.29	0.34	0.23	0.19	0.34
1-Nonanal	0.00025	28.32	16.75	16.72	33.39	39.08	39.72	24.43	32.25	31.56
1-Propanol	38	0.00	0.00	0.00	0.00	0.00	0.00	0.00	0.00	0.00
2-Methyl propanal	0.0013	0.98	2.07	1.72	0.75	0.65	0.74	1.06	0.61	0.54
2-Methylbutanal	0.0002	100.00	100.00	100.00	100.00	100.00	100.00	100.00	100.00	100.00
2-Methylbutanoic acid, methyl ester	0.0004	9.69	6.84	6.13	29.36	24.77	29.39	11.28	10.29	10.81
2-Propanol	0.4	0.05	0.04	0.04	0.05	0.05	0.05	0.05	0.05	0.05
3-Hydroxy-2-butanone	-	-	-	-	-	-	-	-	-	-
3-Methyl butanal	0.00032	35.53	55.31	59.30	25.13	16.93	20.74	29.66	23.06	22.93
6-Methyl-5-hepten-2-one	0.05	0.18	0.19	0.16	0.28	0.38	0.46	0.25	0.34	0.26
Acetaldehyde	0.0027	1.78	0.85	0.68	1.48	0.83	0.90	1.06	0.64	0.77
Acetic acid ethyl ester	18	0.00	0.00	0.00	0.00	0.00	0.00	0.00	0.00	0.00
Acetic acid propyl ester	4.4	0.00	0.00	0.00	0.00	0.00	0.00	0.00	0.00	0.00
Acetone	120	0.00	0.00	0.00	0.00	0.00	0.00	0.00	0.00	0.00
Acetonitrile	67	0.00	0.00	0.00	0.00	0.00	0.01	0.00	0.00	0.00
Butanal	8.8	0.00	0.00	0.00	0.00	0.00	0.00	0.00	0.00	0.00
Cyclohexanone	0.21	0.01	0.01	0.00	0.01	0.01	0.01	0.01	0.01	0.01
Dimethyl sulfide	0.0025	12.43	7.28	4.49	11.52	11.00	11.97	10.27	11.57	11.29
Ethanol	0.17	0.74	0.34	0.21	1.24	1.27	1.27	0.89	1.03	0.96
Furan	28	0.00	0.00	0.00	0.00	0.00	0.00	0.00	0.00	0.00
Hexanal	0.0045	1.76	0.97	0.51	8.48	5.44	6.06	1.43	1.61	1.60
Isoprene	0.001	66.48	33.73	22.06	83.27	70.33	74.59	40.72	54.09	53.73
Methanol	131	0.00	0.00	0.00	0.00	0.00	0.00	0.00	0.00	0.00
Methyl acetate	151	0.00	0.00	0.00	0.00	0.00	0.00	0.00	0.00	0.00
Methyl propionate	0.00072	8.15	6.29	3.73	7.92	8.00	7.93	8.11	7.21	9.41
Propanal	0.0026	8.16	5.18	2.99	29.22	20.43	22.52	7.83	7.71	7.79
Tetrahydrofuran	10.2	0.00	0.00	0.00	0.00	0.00	0.00	0.00	0.00	0.00

Relative Odor Activity Values (ROAV) of volatile compounds detected by GC-IMS during oral processing of potato chips. ROAV was calculated using 2-methylbutanal as the reference (ROAV = 100). ROAV ≥1 identifies candidate aroma compounds for further analysis. Threshold values were sourced from Gemert (2011). "-" indicates not detected.

### 3.3 Morphology modulates the retronasal aroma pathway

**Fig. 5:**
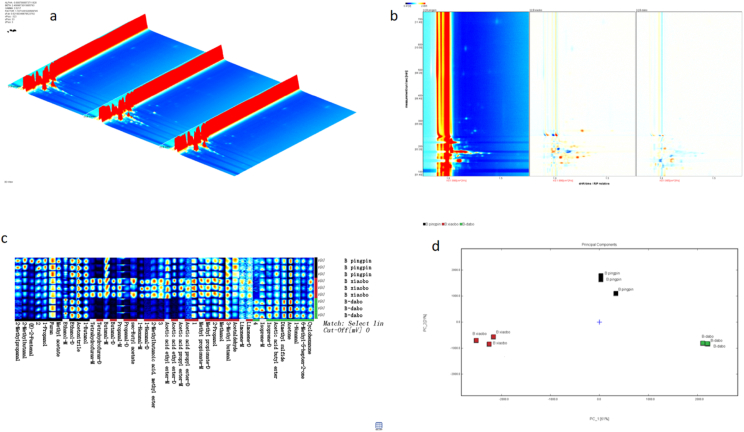
GC-IMS flavor fingerprints of potato chips with different slice morphologies during retronasal processing. a:Three-dimensional topographic of volatile compounds of different slice morphologies. b:Two-dimensional GC-IMS spectrum of the potato chips. c: volatile flavor compound profiles of potato chips with different slice morphologies during retronasal processing. Each row represents all selected signal peaks from a sample, while each column indicates the differences in signal intensity of the same compound across different samples. darker color signifies a higher compound concentration and stronger signal intensity. "-M″ and "-D″ denote monomer and dimer, respectively. Section A illustrates the variations in volatile flavor compounds of flat chips during retronasal processing. Section B displays the variations in volatile flavor compounds of fine rippled chips during retronasal processing. Section C presents the variations in volatile flavor compounds of deep rippled chips during retronasal processing. d: Principal component analysis of volatile compounds in potato chips with different slice morphologies during retronasal processing.

Given that retronasal olfaction accounts for 80-95% of flavor perception (Pu et al., 2022), we specifically analyzed volatiles released via this critical pathway. GC-IMS monitoring of retronasal aroma revealed a total of 30 VOCs (Table 5, Fig. 5), whose release was distinctly shaped by chip morphology (Table 6).

**Table 5:** The volatile compounds during potato chips retronasal processing detected by GC-IMS.

N.O.	Compound	CAS	Formula	Retention index	Retention time	drift time	Odor characteristics
1	Acetone	C67641	C3H6O	819	143.41	1.11	fresh, apple, pear
2	Ethanol-M	C64175	C2H6O	944.2	179.46	1.05	aromaticity
3	Ethanol-D	C64175	C2H6O	941.8	178.55	1.13	aromaticity
4	Butanal-M	C123728	C4H8O	884.9	159.97	1.09	pungent, fruity, green leaf
5	Butanal-D	C123728	C4H8O	885.6	160.17	1.28	pungent, fruity, green leaf
6	Acetic acid ethyl ester-M	C141786	C4H8O2	902.7	164.76	1.11	fresh, fruity, sweet, grassy
7	Acetic acid ethyl ester-D	C141786	C4H8O2	898.6	163.66	1.33	fresh, fruity, sweet, grassy
8	Methyl acetate	C79209	C3H6O2	794.2	137.64	1.20	Ester, Green)
9	2-Methylbutanal	C96173	C5H10O	925.3	172.23	1.40	almond, cocoa, malt
10	Methyl propionate-M	C554121	C4H8O2	927.7	173.13	1.09	Fruit, Rum
11	Methyl propionate-D	C554121	C4H8O2	927.4	173.03	1.33	Fruit, Rum
12	2-Propanol	C67630	C3H8O	928.2	173.33	1.22	alcohol, spicy
13	Acetic acid propyl ester-M	C109604	C5H10O2	987.2	197.05	1.16	fruity, pear
14	Acetic acid propyl ester-D	C109604	C5H10O2	986.9	196.92	1.48	fruity, pear
15	sec-Butyl acetate	C105464	C6H12O2	987.4	197.13	1.22	Fruity
16	2-Methylbutanoic acid, methyl ester	C868575	C6H12O2	998.6	202.24	1.19	apple
17	Acetonitrile	C75058	C2H3N	1022.5	215.03	1.04	floral
18	1-Propanol	C71238	C3H8O	1047.1	229.10	1.11	alcohol, pungent
19	1-Butanol	C71363	C4H10O	1151.1	309.88	1.17	wine
20	Cyclohexanone	C108941	C6H10O	1294.7	487.04	1.15	strong pungent, earthy
21	6-Methyl-5-hepten-2-one	C110930	C8H14O	1351.5	585.09	1.18	citrus, fruity, mouldy, ketone
22	Limonene-M	C138863	C10H16	1201.2	363.58	1.22	lemon, sweet, orange, pine oil
23	Limonene-D	C138863	C10H16	1200.1	362.34	1.29	lemon, sweet, orange, pine oil
24	Methanol	C67561	CH4O	905	165.38	1.03	alcohol, pungen
25	3-Methyl butanal	C590863	C5H10O	938.6	177.31	1.18	chocolate, fat
26	Isoprene-M	C78795	C5H8	670.2	112.08	1.06	oil
27	Isoprene-D	C78795	C5H8	675.2	113.01	1.22	oil
28	Propanal-M	C123386	C3H6O	782.5	135.00	1.07	pungent, green grassy
29	Propanal-D	C123386	C3H6O	779.2	134.26	1.14	pungent, green grassy
30	Acetaldehyde	C75070	C2H4O	734	124.56	1.09	green, slight fruity
31	Tetrahydrofuran-M	C109999	C4H8O	874.3	157.18	1.06	ether
32	Tetrahydrofuran-D	C109999	C4H8O	873.6	156.99	1.23	ether
33	1-Hexanal-M	C66251	C6H12O	1098.3	261.62	1.27	fresh, green, fat, fruity
34	1-Hexanal-D	C66251	C6H12O	1096.4	260.13	1.56	fresh, green, fat, fruity
35	Acetic acid butyl ester	C123864	C6H12O2	1083.3	251.50	1.23	fruity
36	1-Nonanal	C124196	C9H18O	1401.9	688.60	1.48	rose, citrus, strong oily
37	(E)-2-Pentenal	C1576870	C5H8O	1134.1	293.43	1.11	potato, peas
38	2-Methylpropanal	C78842	C4H8O	791.5	137.03	1.28	banana, melon, slightly nutty
39	Furan	C110009	C4H4O	856.2	152.55	0.94	special
40	Dimethyl sulfide	C75183	C2H6S	749.8	127.88	0.95	cabbage, sulfur, gasoline
41	1	-	-	1022.7	215.15	1.11	
42	2	-	-	932.3	174.89	1.28	
43	3	-	-	905.8	165.60	1.28	
44	4	-	-	966	188.20	1.26

**Table 6:** Differences in volatile flavor compound concentrations of potato chips with different slice morphologies during retronasal processing.

	A-10s	B-10s	C-10s
(E)-2-Pentenal	0.31 ± 0.22^a^	0.25 ± 0.02^a^	0.21 ± 0.05a
1-Butanol	0.37 ± 0.04^a^	0.36 ± 0.02^a^	0.3 ± 0.04a
1-Hexanal	0.53 ± 0.02^b^	1.91 ± 0.09^a^	0.49 ± 0.03b
1-Nonanal	1.13 ± 0.09^a^	0.87 ± 0.11^b^	1.28 ± 0.09a
1-Propanol	0.48 ± 0.02^a^	0.42 ± 0.01^b^	0.28 ± 0.01c
2-Methylbutanal	0.62 ± 0.16^a^	0.42 ± 0.01^ab^	0.27 ± 0.02b
2-Methylbutanoic acid, methyl ester	0.23 ± 0.02^b^	0.59 ± 0.04^a^	0.14 ± 0.02c
2-Methylpropanal	0.07 ± 0.01^a^	0.05 ± 0.01^b^	0.05 ± 0.01 ab
2-Propanol	1.2 ± 0.08^b^	1.37 ± 0.04^a^	0.85 ± 0.03c
3-Methyl butanal	2.27 ± 0.2^ab^	2.44 ± 0.03^a^	2.02 ± 0.09b
6-Methyl-5-hepten-2-one	0.49 ± 0.08^a^	0.31 ± 0.08^b^	0.56 ± 0.04a
Acetaldehyde	0.08 ± 0.03^a^	0.08±0^a^	0.07 ± 0.01a
Acetic acid butyl ester	0.17 ± 0.02^a^	0.16 ± 0.02^a^	0.19 ± 0.02a
Acetic acid ethyl ester	1.57 ± 0.16^b^	2.04 ± 0.01^a^	0.67 ± 0.01c
Acetic acid propyl ester	0.51 ± 0.07^b^	1.06 ± 0.05^a^	0.45 ± 0.03b
Acetone	24.5 ± 0.75^b^	23.11 ± 0.2^b^	28.03 ± 1.48a
Acetonitrile	24.18 ± 0.47^a^	24.27 ± 0.42^a^	21.73±2a
Butanal	1.02 ± 0.08^b^	1.48 ± 0.09^a^	0.35 ± 0.03c
Cyclohexanone	0.29 ± 0.01^a^	0.29 ± 0.02^a^	0.3 ± 0.01a
Dimethyl sulfide	4.33 ± 0.22^b^	4.07 ± 0.11^b^	5.03 ± 0.25a
Ethanol	21.48 ± 0.76^b^	15.08 ± 0.21^c^	23.34 ± 0.51a
Furan	1.59 ± 0.06^a^	1.34 ± 0.05^b^	1.5 ± 0.06a
Isoprene	1.51 ± 0.12^b^	1.64 ± 0.04^b^	1.97 ± 0.1a
Limonene	0.27 ± 0.01^a^	0.26 ± 0.06^a^	0.28 ± 0.03a
Methanol	4.12 ± 0.15^a^	4.62 ± 0.29^a^	4.08 ± 0.37a
Methyl acetate	0.34 ± 0.03^a^	0.34 ± 0.01^a^	0.15 ± 0.02b
Methyl propionate	1.89 ± 0.06^b^	2.34 ± 0.07^a^	1.69 ± 0.01c
Propanal	0.76 ± 0.03^b^	1.79 ± 0.05^a^	0.78 ± 0.05b
sec-Butyl acetate	0.1 ± 0.01^b^	0.33 ± 0.11^a^	0.09 ± 0.01b
Tetrahydrofuran	0.92 ± 0.03^b^	3.84 ± 0.5^a^	0.75 ± 0.01b
1	1.22 ± 0.04^b^	1.65 ± 0.04^a^	1.07 ± 0.12b
2	1.35 ± 0.15^a^	1.11 ± 0.01^ab^	0.92 ± 0.08b
3	0.06 ± 0.01^b^	0.11± 0.01^a^	0.02±0c
4	0.04±0^b^	0.03 ± 0.02^b^	0.07 ± 0.01a

Different letters in the same row means the significant differences of contents in potato chips caused as oral processing (P < 0.05). The contents were expressed as the average ± standard deviation.

As a qualitative screening, ROAV analysis of retronasal volatiles (Table 7) identified several compounds with potential sensory relevance. For example, 1-propanol showed higher relative abundance in fine-corrugated chips. However, consistent with our methodological approach, the primary interpretation of aroma contribution was based on the dynamic release kinetics of these compounds across the mastication timeline (Table 9) and their alignment with the TDS sensory profiles. Our analysis of the retronasal pathway extends beyond merely confirming the presence of volatile compounds; it reveals that morphological engineering can program the final olfactory signal input. We found that different chip morphologies not only altered the release kinetics of volatiles in the oral cavity but, more importantly, exerted differential control over the composition and temporal sequence of volatiles reaching the olfactory receptors via the retronasal route.

**Table 7:** Relative odor activity values (ROAVs) of key volatile flavor compounds.

Compounds	Threshold mg/L	ROAV		
		A-10s	B-10s	C-10s
(E)-2-Pentenal	0.0005	0.00	0.00	0.00
1-Butanol	0.01	0.01	0.01	0.00
1-Hexanal	0.0045	0.00	0.00	0.00
1-Nonanal	0.00025	0.00	0.00	0.00
1-Propanol	38	17.78	20.55	13.54
2-Methylbutanal	0.0002	0.00	0.00	0.00
2-Methylbutanoic acid, methyl ester	0.0004	0.00	0.00	0.00
2-Methylpropanal	0.0013	0.00	0.01	0.00
2-Propanol	0.4	0.07	0.07	0.05
3-Methyl butanal	0.00032	0.00	0.00	0.00
6-Methyl-5-hepten-2-one	0.05	0.02	0.04	0.01
Acetaldehyde	0.0027	0.01	0.01	0.00
Acetic acid butyl ester	0.066	0.09	0.09	0.03
Acetic acid ethyl ester	18	2.56	1.99	2.69
Acetic acid propyl ester	4.4	1.92	0.94	0.97
Acetone	120	1.10	1.17	0.43
Acetonitrile	67	0.62	0.62	0.31
Butanal	8.8	1.94	1.34	2.55
Cyclohexanone	0.21	0.16	0.16	0.07
Dimethyl sulfide	0.0025	0.00	0.00	0.00
Ethanol	0.17	0.00	0.00	0.00
Furan	28	3.95	4.70	1.86
Isoprene	0.001	0.00	0.00	0.00
Limonene	0.0175	0.01	0.02	0.01
Methanol	131	7.13	6.37	3.21
Methyl acetate	151	100.00	100.00	100.00
Methyl propionate	0.00072	0.00	0.00	0.00
Propanal	0.0026	0.00	0.00	0.00
sec-Butyl acetate	0.0089	0.02	0.01	0.01
Tetrahydrofuran	10.2	2.47	0.60	1.35

Relative Odor Activity Values (ROAV) of key volatile flavor compounds detected during retronasal processing. ROAV was calculated using methyl acetate as the reference (ROAV = 100), ROAV ≥1 identifies candidate aroma compounds for further analysis. Threshold values were sourced from Gemert (2011). "-" indicates not detected.

Specifically, fine-corrugated chips promoted the rapid release of aldehydes and esters. Retronasal data indicate that these compounds, which impart the initial "top notes" of fried and fruity aromas, reached the olfactory receptors earlier and more intensely. In contrast, the deep-corrugated structure retained alcohols and ketones, implying a prolonged signal duration for compounds that often contribute "base notes" or creamy flavors. This establishes a solid theoretical foundation for developing next-generation foods with customized flavor experiences, particularly for the efficient utilization of limited flavoring agents in healthier food products.

### 3.4 The mechanistic bridge: linking texture properties to flavor release

We postulated that the differential release kinetics originated from the fundamental mechanical properties imparted by each morphology. Texture Profile Analysis confirmed that morphology significantly altered mechanical strength (p < 0.05, Table 8). Deep-corrugated chips exhibited the highest hardness (3.90 N) and fracture force (3.67 N), while flat chips were the most fragile.

**Table 8:** Textural properties of potato chips with different slice morphologies.

	Fracture/N	Hardness/N
A	1.82 ± 0.38^c^	1.83 ± 0.39^c^
B	2.24 ± 0.49^b^	2.27 ± 0.50^ab^
C	3.67 ± 1.40^a^	3.90 ± 1.60^a^

Different letters in the same column means the significant differences of contents in potato chips caused as oral processing (P < 0.05). The contents were expressed as the average ± standard deviation.

**Table 9:** Release Kinetic Parameters of Key Flavor Compounds from Potato Chips with Different slice morphologies.

Compound	Chip Type	AUC	Cmax	Tmax/s
(E)-2-Pentenal	A	2.15 ± 0.20c	0.26 ± 0.05c	5
	B	3.88 ± 0.45a	0.44 ± 0.05a	10
	C	2.58 ± 0.40b	0.32 ± 0.03b	5
Dimethyl sulfide	A	25.23 ± 0.95a	2.79 ± 0.07a	10
	B	19.21 ± 0.35c	1.93 ± 0.08a	5
	C	25.41 ± 0.30b	2.60 ± 0.15a	5
Hexanal	A	5.97 ± 0.35b	0.68 ± 0.05b	5
	B	19.29 ± 0.85a	2.55 ± 0.19a	5
	C	6.40 ± 0.20b	0.65 ± 0.03b	5
2-Methylbutanoic acid, methyl ester	A	3.94 ± 0.15c	0.42 ± 0.01c	10
	B	7.34 ± 0.20a	0.79 ± 0.03a	5
	C	3.90 ± 0.25b	0.46 ± 0.04b	5
Methyl propionate	A	5.83 ± 0.10a	0.69 ± 0.04a	10
	B	3.88 ± 0.20a	0.40 ± 0.01a	10
	C	5.28 ± 0.15a	0.61 ± 0.03a	15
Propanal	A	18.08 ± 0.20 ab	2.07 ± 0.06 ab	10
	B	40.62 ± 0.35a	5.09 ± 0.22a	5
	C	18.42 ± 0.20 ab	2.06 ± 0.12 ab	5

Different letters in the same column indicate significant differences in AUC and Cmax values among chip morphologies (P < 0.05). Tmax values are reported as the time point at which Cmax was observed, and should be interpreted as trends rather than precise kinetic parameters due to the limited temporal resolution.

These textural parameters directly dictated oral processing. The superior mechanical strength of corrugated chips necessitated a more progressive, step-wise fracture pattern, as opposed to the catastrophic breakdown of flat chips. This slower fracture kinetics effectively trapped volatile compounds within larger matrix fragments for longer, leading to the gradual release profile observed by GC-IMS in both oral and retronasal phases. This establishes a clear pathway: morphology → mechanical strength → fracture behavior → flavor release kinetics. At 5 s of chewing, the fragment size of deep-corrugated chips was significantly larger than that of flat and fine-corrugated chips, indicating that the corrugated structure enhances mechanical strength and reduces the degree of fragmentation during the early stage of mastication. A larger fragment size implies a smaller specific surface area and fewer fracture interfaces, thereby slowing the release of internal volatile compounds. This directly reflects the “physical micro-reservoir” mechanism: the corrugated structure inhibits rapid disintegration, thereby retaining aroma compounds within larger fragments and achieving sustained release. By 15 s of chewing, the fragment sizes of the three groups tended to converge (ranging from 2.54 to 3.10 mm), which is consistent with the GC-IMS results showing that concentrations of compounds such as hexanal tended to level off in the later stage, further corroborating the regulatory role of fragment size on release kinetics.

**Fig. 6:**
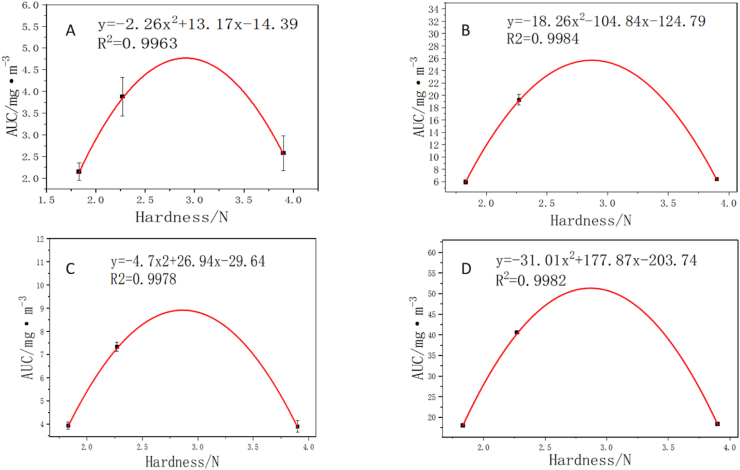
Correlation analysis between potato chip hardness and the total release (AUC) of key flavor compounds. (A) (E)-2-Pentenal; (B) Hexanal; (C) 2-Methylbutanoic acid, methyl ester; (D) Propanal. Quadratic regression lines are shown to visualize trends, but should be interpreted with caution as they are based on only three data points (n = 3). The observed trends suggest a potential non-monotonic relationship within the tested hardness range.

An interesting observation was the quadratic trend (R^2^ > 0.99) between chip hardness and the total release (AUC) of certain flavor compounds such as (E)-2-pentenal and hexanal (Fig. 6). However, this curve fitting is based on only three data points (n = 3), representing the three chip morphologies. The release appeared to be maximized at an intermediate hardness (approximately 2.3 N, representative of fine-corrugated chips) and diminished in both simpler and more complex structures. This finding should therefore be considered a preliminary observation specific to the tested system and hardness range, rather than a robustly validated model. The observed trend suggests the possibility of an optimal mechanical strength window for volatile release, which may be related to fragment size and specific surface area—a hypothesis that warrants further investigation with a broader range of samples. Future studies incorporating a wider range of chip morphologies and hardness values are needed to validate the nature of this relationship.

**Fig. 7:**
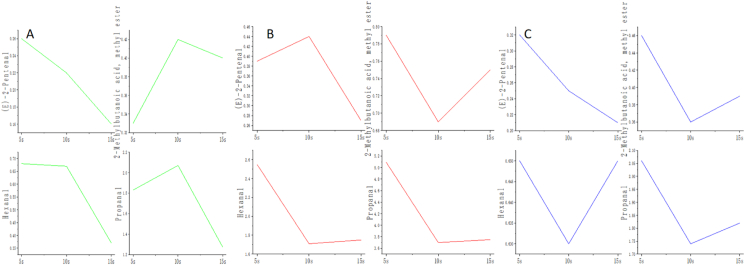
Release kinetics of key aroma compounds from potato chips with different morphologies during oral processing. (A) Flat, (B) Fine corrugated, and (C) Deep corrugated.

### 3.5 Methodological considerations and limitations

It is important to acknowledge the limitations of the simulated oral processing conditions employed in this study. The chewing frequency (1.5 Hz) and saliva flow rate (1.5 mL/min) were selected based on average values reported in the literature to ensure standardized and reproducible conditions (Luckett and Seo, 2017). However, these parameters do not account for the significant inter-individual variability in natural mastication patterns, which can be influenced by factors such as age, gender, and oral physiology. Such variability could affect the absolute magnitude of aroma release. Nevertheless, this study focuses on the relative differences in release kinetics between chip morphologies under controlled conditions, and the standardized protocol effectively minimizes confounding variables, allowing for a valid comparison of the structural effects. A further methodological consideration relates to the temporal resolution of the GC-IMS analysis, which relied on three discrete time points (5, 10, and 15 s). While this approach captured the general trends of aroma release, it may not be sufficient to precisely determine kinetic parameters such as T_max_, particularly for compounds with rapid initial release dynamics. Therefore, the conclusions regarding kinetic parameters should be interpreted with caution, focusing on overall patterns rather than precise values.

Future studies should consider incorporating a broader range of chewing parameters, utilizing in vivo mastication monitoring, and employing higher temporal resolution sampling to better capture the complexity of individual oral processing behaviors and dynamic aroma release.

## 4 Conclusions

This study mechanistically deciphers how potato chip morphology dictates flavor perception by establishing a comprehensive "structure–release–perception" linkage, with a particular emphasis on the critical role of the retronasal pathway. The main findings are.

(1) Chip morphology could influence flavor release kinetics by determining mechanical strength and fracture behavior. Corrugated structures function as physical micro-reservoirs, imparting a significant sustained-release effect on key aroma compounds during oral processing.
(2) An observed quadratic trend between hardness and the AUC suggests a potential non-monotonic relationship, indicating a possible optimal window of mechanical strength for flavor release within the range of chip morphologies tested.
(3) Crucially, this sustained-release effect strategically shapes the olfactory signal delivered via the retronasal pathway. Morphology was found to selectively modulate the release of specific volatile classes (e.g., aldehydes/esters vs. alcohols/ketones), thereby programming the temporal narrative and composition of the aroma profile that ultimately reaches the olfactory receptors.
(4) Consequently, this engineered release kinetics is associated with prolonged crispness and enhanced flavor persistence in sensory evaluation, as evidenced by TDS analysis.

In summary, morphological engineering is a potent, non-chemical strategy for tailoring temporal flavor delivery. This work provides a theoretical foundation and a practical framework for optimizing sensory properties in starchy snacks and related food systems through rational structural design, opening avenues for creating customized eating experiences without altering product formulation.

## Informed consent

Informed consent was obtained from all individual participants included in the study.

## Ethical approval

All procedures performed in studies involving human participants were in accordance with the ethical standards of the institutional and/or national research committee and with the 1964 Helsinki declaration and its later amendments or comparable ethical standards. The study was approved by the Academic Committee of the Institute of Economic Crops, Yunnan Academy of Agricultural Sciences.

## Authors’ contribution

The experiment was designed by Dr. Ying Wang. She analyzed data, and wrote the manuscript. Prof. Wanlin Yang, Prof. Qiongfen Yang supervised the research and reviewed the manuscript. Dr. Jitian He, Dr Fankui Zeng contributed in data collection and arrangement. Dr Xianping Li and Yuanxing Liu provided the potato chip samples. Dr Wenjing Huang was responsible for E-nose data analysis, Dr Chunyan Zhang was responsible for GC-IMS data analysis.

## Availability of data and materials

The datasets and materials will be provided upon publication.

## Ethics approval and consent to participate

The Academic Committee of the Industrial Crops Research Institute at the Yunnan Academy of Agricultural Sciences granted ethical approval for this study.

## Consent for publication

All authors agreed to publish this manuscript.

## Declaration of competing interest

The authors declare the following financial interests/personal relationships which may be considered as potential competing interests:Ying Wang reports financial support was provided by Yunnan Academy of Agricultural Sciences. If there are other authors, they declare that they have no known competing financial interests or personal relationships that could have appeared to influence the work reported in this paper.
