# Supplementary material for: Structural design dictates flavor release kinetics in potato chips: Unraveling the mechanism by integrated temporal sensory and chemical analysis
Source: Curr Res Food Sci. 2026 May 2;12:101432. doi: 10.1016/j.crfs.2026.101432 (PMC13156765; doi:10.1016/j.crfs.2026.101432)
Supplement: Multimedia component 1 [file mmc1.docx]

Supplement table 1

| Sample | Average particle size after 5 s of chewing/mm | Average particle size after 10 s of chewing/mm | Average particle size after 15 s of chewing/mm |
| --- | --- | --- | --- |
| Flat | 5.26 ± 2.29c | 4.65 ± 1.42 b | 2.54 ± 1.67 b |
| Fine-corrugated | 7.06 ± 3.59 b | 4.69 ± 1.62b | 2.83 ± 1.61b |
| Deep-corrugated | 9.99 ± 2.83a | 5.46 ± 2.04a | 3.10 ± 1.34a |

Different letters in the same column indicate significant differences in mean particle size among different chip morphologies after the same chewing time (P < 0.05), with 30 replicates per sample per chewing time.
